# Resveratrol-Loaded Polydimethylsiloxane–Silica Hybrid Materials: Synthesis, Characterization, and Antitumoral Activity

**DOI:** 10.3390/polym16070879

**Published:** 2024-03-22

**Authors:** Sofia Viegas, Diogo Marinheiro, Verónica Bastos, Ana L. Daniel-da-Silva, Ricardo Vieira, Helena Oliveira, José Carlos Almeida, Bárbara J. M. L. Ferreira

**Affiliations:** 1 Department of Chemistry, CICECO-Aveiro Institute of Materials, University of Aveiro, 3810-193 Aveiro, Portugal; 2 Department of Biology, CESAM-Centre for Environmental and Marine Studies, University of Aveiro, 3810-193 Aveiro, Portugalholiveira@ua.pt (H.O.); 3 Department of Materials and Ceramic Engineering, CICECO-Aveiro Institute of Materials, University of Aveiro, 3810-193 Aveiro, Portugal

**Keywords:** bone tissue, osteosarcoma, sol–gel hybrid materials, PDMS-SiO_2_ system, resveratrol, drug delivery

## Abstract

In this work, hybrid materials within the polydimethylsiloxane–silica (PDMS–SiO_2_) system, synthesized via the sol–gel method, were developed and characterized for their potential to incorporate and release the bioactive compound resveratrol (RES). RES was incorporated into the materials with a high loading efficiency (>75%) using the rotary evaporator technique. This incorporation induced the amorphization of RES, resulting in enhanced solubility and *in vitro* release when compared to the free polyphenolic compound. The release profiles displayed pH dependence, exhibiting notably faster release at pH 5.2 compared to pH 7.4. The gradual release of RES over time demonstrated an initial time lag of approximately 4 h, being well described by the Weibull model. *In vitro* cytotoxicity studies were conducted on human osteosarcoma cells (MG-63), revealing a concentration-dependent decrease in cell viability for RES-loaded samples (for concentrations >50 µg mL^−1^).

## 1. Introduction

The first two decades of this new millennium witnessed the emergence of developing biomaterials aimed at bone diseases [1], prompted by the rise in human longevity and the subsequent increase in chronic diseases such as osteoporosis [2], osteoarthritis [3], or cancers such as osteosarcoma [4]. While bones possess a natural regenerative capacity, significant defects exceeding double the bone diameter may lead to scar tissue formation or persistent weaknesses, necessitating clinical intervention. Current approaches such as bone autografts, allografts, and synthetic bone substitutes [5,6] have limitations including high costs, transplantation risks, and material durability concerns [7]. To address these limitations, there is a growing interest in biomaterials designed not only to fulfil structural functions by replacing damaged tissue, but also to actively contribute to tissue regeneration [8,9]. In this context, organically modified silicates (ORMOSILs), a family of organic–inorganic hybrid materials obtained through the sol–gel method, have gained prominence. These have gained much interest from the scientific community, due to their wide range of applications, such as biomaterials, coatings, scaffolds, or drug delivery systems (DDSs) [10]. Additionally, these modified silicates demonstrate great potential in the field of bone tissue engineering [11,12]. One of the most studied types of ORMOSILs is formed through the reaction between tetraethyl orthosilicate (TEOS) and polydimethylsiloxane (PDMS) [13]. PDMS is considered a polymeric material belonging to the subclass of elastomers, and its varied characteristics (i.e., chemical and thermal stability [14,15], biocompatibility [16,17], flexibility [18], among others) make it one of the most used silicon-based polymers [18,19]. ORMOSILs, particularly those formed by the reaction between tetraethyl orthosilicate (TEOS) and polydimethylsiloxane (PDMS), show promise in various applications, including biomaterials [20] and drug delivery systems (DDSs) [21,22], and have demonstrated potential in bone tissue engineering [23,24]. The incorporation of the organic component into the inorganic network along with the interactions between both components allows the obtention of hybrid materials endowed with desirable morphology, size, and multifunctional properties [25,26]. 

Resveratrol (3,5,4′-trihydroxystilbene; RES) is a stilbene polyphenol, with two phenol rings linked to each other by an ethylene bridge [27,28]. Its chemical structure has two conformations, *cis*- and *trans*-resveratrol, with the *trans* conformation being predominant and associated with various beneficial biological activities [29]. The pharmacological interest in *trans*-RES emerged from studies identifying its presence in wine [30], linking its consumption to health benefits, giving rise to the so-called “French Paradox” [31]. Subsequent research has highlighted RES’s diverse biological properties, including antioxidant, cardioprotective, neuroprotective, anti-inflammatory, and anticancer/antitumoral activities [32,33]. Several studies indicate that RES can positively impact bone healing and regeneration, due to its osteogenic and osteoinductive properties [34,35]. Its potential benefits in specific bone related-diseases, such as enhancing bone mineral density in osteoporosis patients [36,37] and its potential in the treatment of osteosarcoma [38,39,40], further underscore its therapeutic significance. Despite its immense potential, RES faces pharmacokinetic limitations including low water solubility affecting bioavailability [41,42], a short biological half-life [43,44], and chemical instability due photosensitivity and susceptibility to oxidation [45]. Through the years, researchers have developed diverse DDSs, aiming not only to increase RES absorption but also to overcome solubility, stability, and bioavailability issues, while protecting against light, oxygen, and other environmental factors that can compromise its properties [46]. Some examples of such materials encompass cyclodextrin complexes [47,48], liposomes [49,50], polymeric micelles [51], or nanoparticles [52,53]. Nonetheless, these DDSs are far from perfect, presenting limitations such as low stability, limited drug loading, the need for organic solvents, and high production costs [54]. More recently, several studies have reported the successful encapsulation of RES in silica-based materials with potential for various clinical applications [55,56,57]. However, to the best of the authors’ knowledge, there are no reports regarding the incorporation of RES into PDMS-SiO_2_ hybrid materials. Therefore, the present work aimed to prepare a novel RES-loaded hybrid material that combines the physicochemical properties of the PDMS-SiO_2_ system with the biological properties of RES and evaluate its therapeutic potential as a DDS for osteosarcoma. 

## 2. Materials and Methods

### 2.1. Chemicals

Tetraethyl orthosilicate (TEOS, ≥99.0%), polydimethylsiloxane silanol terminated (PDMS, 550 g·mol^−1^ average molecular weight), phosphate buffered saline at pH 7.4 (0.01 M PBS, 25 °C) packets, and the dialysis cellulose tubing membrane (3 kDa MW cut-off) were obtained from Sigma-Aldrich (St. Louis, MO, USA). Isopropyl alcohol (IPA, ≥99.8%) was acquired from Fisher Chemical (Loughborough, UK). Hydrochloric acid (HCl, 25%) was purchased from Panreac Quimica SLU (Barcelona, Spain), *trans*-resveratrol (RES, 99%) from Tokyo Chemical Industry (TCI Europe N.V., Zwijndrecht, Belgium), and ethanol absolute from VWR (Radnor, PA, USA). Ultrapure water (Mili-Q^®^) was used in all experiments. All chemicals were used as received without further purification.

### 2.2. Synthesis of the Hybrid Materials

The materials have been prepared through a sol–gel process at room temperature, by sequentially adding IPA, PDMS, H_2_O, HCl, and TEOS, following the molar ratios detailed in Table 1. 

First, IPA, PDMS, H_2_O, HCl, and TEOS were introduced in this order, while continuously stirring with a magnetic stirrer for 30 min. HCl served as a catalyst for the hydrolysis/condensation reactions. Subsequently, the resulting suspension was transferred to a petri dish and allowed to age at room temperature for 24 h, remaining liquid and transparent. Later, the samples were placed in an oven at 60 °C for 24 h. Finally, to eliminate residual solvents, the samples underwent further treatment in an oven at 150 °C for 1 h, resulting in monolithic specimens (Figure S1, Supplementary Material). A schematic representation of the synthesis procedure can be found in Figure S2, Supplementary Material. Samples were ground to fine powders using a mortar and pestle of agate and then sieved to obtain particles in the range of 0.180–0.355 mm size. The sieves used were from Filtra Vibration with a diameter of 200 mm. Notably, sample M0C0-II resulted in a transparent but non-homogenous material, suggesting that the absence of HCl adversely influenced the hydrolysis/condensation processes and consequently the final material. Consequently, this sample was excluded from subsequent stages of this study.

### 2.3. Incorporation of RES

RES incorporation was performed using the rotary evaporation technique [55,57]. In a typical procedure, 160 mg of RES was dissolved in 30 mL of ethanol and sonicated for 4 min. Afterwards, 200 mg of the PDMS-SiO_2_ material was added to the solution and the dispersion was placed in the ultrasonic bath for 10 min. The solvent was then gently evaporated at 50 °C using a rotary evaporator (Buchi) until all ethanol was removed, to obtain the RES-loaded PDMS-SiO_2_ samples (-RES). The final solids were scraped off from the round bottom flask and stored at room temperature, protected from light with aluminum foil.

Loading efficiency was determined using Equation (1). The loading capacity of the materials was determined by TGA and it corresponds to the RES content of each sample, and the theoretical loading capacity corresponds to the loading capacity if all the RES used had been loaded onto the materials.

(1) $$Loading\;efficiency\left(\%\right)=\frac{Loading\;capacity}{Theoretical\;loading\;capacity}\times 100$$

### 2.4. Physicochemical Characterization of the Materials

Powder samples were subjected to structural and microstructural characterization. 

#### 2.4.1. X-ray Diffraction (XRD)

X-ray diffractograms were recorded using a PANalytical-X’pert-PRO diffractometer (Malvern PANalytical, Malvern, UK) with Kα(Cu) radiation and a curved graphite monochromator in the range 2° < 2θ < 60°, with a step size of 0.0263° and a step time of 96.39 s, at room temperature. 

#### 2.4.2. Fourier Transform Infrared Spectroscopy (FTIR)

FTIR spectra were collected on a Mattson–7000 spectrometer (Mattson Instruments Inc., Madison, WI, USA), and 128 scans were collected per sample over the range of 4000 to 300 cm^−1^ at a resolution of 2.0 cm^−1^, employing the KBr technique for sample preparation. 

#### 2.4.3. Surface Area Determination by Nitrogen Adsorption

Nitrogen adsorption–desorption isotherms were measured on a Quantachrome-Autosorb IQ2 equipment (Anton Paar USA Inc., Ashland, VA, USA). All samples were degassed at 150 °C for 12 h, followed by cooling to room temperature before measurements. The pore size was calculated from desorption branches of isotherms using the Barrrett–Joyner–Halenda (BJH) method. Surface areas were calculated using the Brunauer–Emmett–Teller (BET) method. 

#### 2.4.4. Thermogravimetric Analysis (TGA)

Thermogravimetric analysis (TGA) was employed to quantify the amount of RES loaded on the materials with a Hitachi STA300 instrument (Hitachi, Tokyo, Japan) at a heating rate of 5 °C/min (temperature range 30–800 °C), under a nitrogen/oxygen atmosphere (3:1). 

#### 2.4.5. Scanning Electron Microscopy (SEM)

Microstructural features were examined via scanning electronic microscopy (SEM) using a Hitachi microscope SU-70 model (Hitachi, Tokyo, Japan) with an acceleration voltage of 10 kV. Samples were prepared by fixing the powder samples in an aluminum sample holder with double-sided carbon tape. UV–VIS spectra were obtained in a GBC Cintra 303 spectrophotometer (GBC Scientific Equipment, Victoria, Australia) operating in the range of 200–500 nm.

### 2.5. In Vitro Solubility and Release Assays

#### 2.5.1. Dissolution Studies

Solubility studies were performed using PBS at pH 7.4. The suspensions were prepared by dispersing 10 mg of the RES-loaded materials in 10 mL of PBS. The samples were kept under continuous yet gentle stirring at 37 °C and shielded from light for 24 h. For comparison, the test was also performed using free RES. The concentration of RES was determined employing the Lambert–Beer law, which correlates the absorbance with the concentration of the sample. With this purpose, the calibration curve was established for RES solutions at pH 7.4. For fixed, known concentrations of resveratrol, the absorbance at λ = 304 nm was measured and plotted against the concentration, revealing a good linear correlation (*r*^2^ = 0.99996). 

#### 2.5.2. *In Vitro* Release Studies

*In vitro* release studies were performed using PBS at two different pH values, 7.4 and 5.2. The suspensions were prepared by dispersing the RES-loaded M0C0-IV material (equivalent to 2 mg of free RES) in 1 mL of PBS, introduced into a dialysis membrane (with a 3.5 kDa molecular weight cut-off) and promptly immersed in 49 mL of PBS. The samples were maintained under continuous yet gentle stirring at 37 °C, protected from light. At predetermined time intervals, 1 mL of the sample was withdrawn and immediately replaced with an equal volume of PBS to maintain sink conditions. For comparison, the test was also conducted using free RES. The concentration of RES was calculated based on the Lambert–Beer law, using calibration curves established through the method previously described for solubility studies (*r*^2^ = 0.9992 and *r*^2^ = 0.99996 for pH 5.2 and 7.4). The absence of a peak or strong absorption in the spectral region of 260–287 nm indicates that there was no *cis*-RES or degradation compounds were formed during the RES release studies [58]. The cumulative release percentage of RES was calculated using Equation (2),

(2) $$Cumulative\;release\left(\%\right)=\frac{Volume\;of\;sample\;withdrawn\;(mL)}{Bath\;volume\;(mL)}\times P\left(t-1\right)+Pt$$

where Pt corresponds to the percentage released at the time t and P_(t−1)_ represents the percentage released prior to time t. The data presented in the release curves are an average of replicates.

#### 2.5.3. Kinetics and Release Models

The kinetics and mechanisms of release were performed by analyzing the fractional release of RES with the function of time and fitting the data with three models: the Noyes–Whitney equation applying Fick’s law, the Korsmeyer–Peppas, and the Weibull model. These models have been successfully applied in studying drug delivery systems, including RES release [57,59], and empirical observations have been combined with physics-based models to predict the overall release behavior of simple and complex DDSs, mainly the temporal release of the encapsulated molecule(s) [60]. The choice of the appropriate model depends on the drug type, excipients, and device composition.

The Korsmeyer–Peppas (KP) model is a semi-empirical model based on Fick’s second law of diffusion for short time diffusion, which relates exponential drug release to elapsed time (*t*), as described by Equation (3):

M_t_/M_∞_ = *k*_KP_ · *(t* − *T_i_)^n^* (3)

where M_t_/M_∞_ corresponds to the fractional release of the drug, *k*_KP_ is a constant that incorporates characteristics of the system, T*_i_* represents the lag time before the onset of the dissolution or release process, and *n* is indicative of the drug release mechanism and/or on the geometry of the system. This model is valid for M*_t_*/M_∞_ ˂ 0.6 [61,62,63]. The Weibull model [63,64,65], an empirical model successfully applied to various dissolution or release curves, is expressed in Equation (4):

(4) $$Mt/M\infty =1-e^{\left(-\frac{(t-T{i)}^{\beta }}{\alpha }\right)}$$

where M_t_ and M*_∞_* are the mass of the drug released at time *t*, α defines the time scale of the process, T*_i_* represents the lag time before the onset of the dissolution or release process, and β is the shape parameter that characterizes the curve formed as either of the following: exponential (β = 1), S-shaped with upward curve followed by turning point (β > 1), or a curve with higher initial slope and after that consistent with the exponential (β < 1). 

The values of α and β obtained from the Weibull model allow to determine the dissolution time, T*_d_*, which represents the time interval necessary to reach 63.2% of drug release, as seen in Equation (5):

(5) $${\alpha =T_{d^{\;\beta }}\to T_{d}=e}^{\frac{\log\left(\alpha \right)}{\beta }}$$

### 2.6. Cell Studies

#### 2.6.1. Reagents

Dimethyl sulfoxide (DMSO; ≥99.7%) and 3-(4,5 dimethyl-2-thiazolyl)-2,5-diphenyl tetrazolium bromide (MTT; 98%) were purchased from Sigma-Aldrich (St. Louis, MO, USA). The MG-63 human osteosarcoma cell line was purchased from American Type Culture Collection (ATCC, Rockville, MD, USA). Dulbecco’s Modified Eagle’s Medium, fetal bovine serum (FBS), l-glutamine, and fungizone (250 U mL^−1^) were obtained from Pan Biotech (Aidenbach, Germany) and penicillin–streptomycin (10,000 U mL^−1^) from Grisp (Porto, Portugal). Trypsin–ethylenediaminetetraacetic acid (EDTA) (0.25% trypsin and 1 mM EDTA) was purchased from Gibco, Life Technologies (Grand Island, NY, USA).

#### 2.6.2. Cell Culture and Sample Preparation

Samples M0C0-IV and M0C0-IV-RES were incubated in the medium, under continuous but gentle stirring at 37 °C for 48 h. After 48 h, the extracts were centrifuged, and used to prepare the different concentrations used in the cell viability assay. MG-63 cells were aseptically grown in Dulbecco’s Modified Eagle’s Medium, supplemented with 10% FBS, 2 mM l-glutamine, 1% penicillin–streptomycin (10,000 U mL^−1^) and 1% fungizone (250 U mL^−1^) at 37 ◦C in a humidified atmosphere with 5% CO_2_. After 24 h, cells were observed for confluence and morphology using an inverted phase-contrast Eclipse TS100 microscope (Nikon, Tokyo, Japan). Sub-confluent cells were trypsinized with trypsin–EDTA (0.25% trypsin and 1 mM EDTA) when monolayers reached 80–90% confluence.

#### 2.6.3. *In Vitro* Cell Viability Assay

Cell viability was determined by the colorimetric MTT assay, which is based on the conversion of MTT into purple formazan crystals by living cells [66]. MG-63 cells were seeded in 96-well plates at 4000 cells well^−1^ and incubated at 37 °C in a 5% CO_2_ humidified atmosphere for 24 h for cell adhesion. After 24 h, the medium was replaced with fresh medium containing the following: (a) pristine M0C0-IV extract (0, 50, 100, 200, 300, 350, and 400 µg mL^−1^), (b) RES-loaded M0C0-IV extract (0, 50, 100, 200, 300, 350, and 400 µg mL^−1^), and (c) bulk RES (0, 75, 150, 300, 450, 500, and 600 µM). Cells exposed to the cell culture medium were used as a negative control, and cell viability was measured after 24 h. At the end of the incubation time, the wells were emptied and washed with PBS to remove the remaining particles, and then fresh medium (100 µL) was placed in each well. After that, 50 µL of MTT (1 g L^−1^ in PBS) was added to each well and incubated for 4 h at 37 °C in a 5% CO_2_ humidified atmosphere. Then, the culture medium with MTT was removed and replaced by 150 µL of DMSO and placed in an orbital shaker (for 2 h, protected from light) for formazan crystal solubilization. The sample’s absorbance (Abs) was measured with a BioTek Synergy HT plate reader (Synergy HT Multi-Mode, BioTeK, Winooski, VT, USA) at 570 nm. The percentage of viability was calculated using Equation (6),

(6) $$Viability\left(\%\right)=\frac{Abs\left(570\right)\;of\;samples}{Abs\left(570\right)\;of\;negative\;control}\times 100$$

Statistical analysis was performed by one-way ANOVA, followed by Dunnett and Dunn’s method (as parametric and non-parametric tests), using Sigma Plot 14.0 software (Systat Software Inc., San Jose, CA, USA). 

## 3. Results and Discussion

### 3.1. Characterization of Unloaded Materials

**Figure 1:**
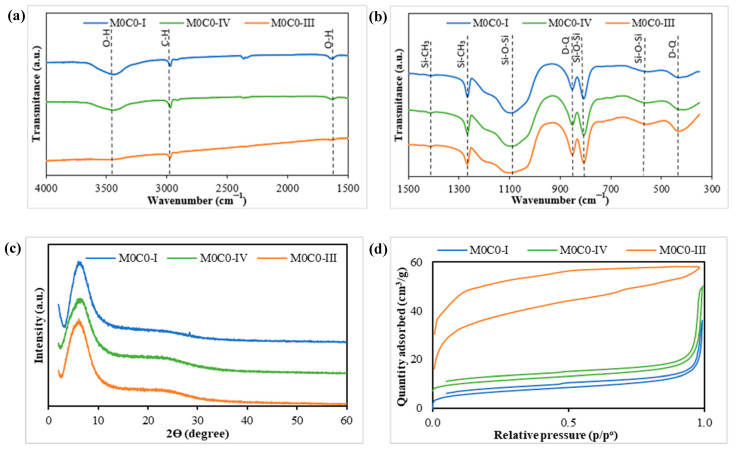
Physicochemical characterization of unloaded materials. (**a**,**b**) FTIR spectra of the PDMS-SiO_2_ samples; (**c**) XRD diffraction pattern of the PDMS-SiO_2_ samples; (**d**) N_2_ adsorption–desorption isotherms of the PDMS-SiO_2_ samples obtained by the BET method.

FTIR spectroscopy studies were performed to examine the chemical bonds present in the unloaded samples (Figure 1a,b). The incorporation of PDMS within the network was confirmed by the presence of asymmetric and symmetric CH_3_ deformation bands at 1408 and 1265 cm^−1^ respectively, along with asymmetrical C-H stretching at 1968 cm^−1^. The formation of the inorganic network composed of Si-O-Si bonds was validated by the appearance of typical asymmetric Si-O-Si stretching vibrations at 1090 cm^−1^, symmetric vibrations of Si-O-Si bonds at 805 cm^−1^, and Si-O-Si vibration in 4-fold siloxane rings at 555 cm^−1^. The presence of bands at 850 cm^−1^ and 430 cm^−1^ are due to the presence of crosslinked SiO_2_ (Q units)-PDMS (D units) structures, thus forming D-Q bonds and confirming the formation of hybrid structures with bonds between the organic and inorganic parts of the material. These values are aligned with those observed in previous works [67,68,69]. Notably, in samples M0C0-I and M0C0-IV, the bands around 430 cm^−1^ exhibit a split into two smaller bands. This is attributed to the lower water content in the preparation, leading to the formation of more hybrid bonds (D-Q structures). In contrast, sample M0C0-III, prepared with a higher amount of water, displays a more defined band at 430 cm^−1^, indicating the presence of less hybrid bonds and longer chains [70]. The IR band observed around 3430 cm^−1^ is likely attributed to hydrogen-bonded silanol groups with absorbed molecular water, while the peak at 1625 cm^−1^ corresponds to O-H bending in the molecular water [68,71]. Additionally, the bands at 2360 cm^−1^ are associated with the asymmetric stretching vibration of the gas-phase CO_2_ originated from ambient air absorption in the optical path outside the FT-IR cell [72].

XRD analysis was performed to confirm the amorphous nature of the PDMS-SiO_2_ samples (M0C0-I, M0C0-III, and M0C0-IV), and the results are presented in Figure 1c. The graph displays a broad peak around a specific 2θ angle, approximately at 6°, which indicates the amorphous nature of the materials. According to the Braggs Law, by determining the 2Ɵ angle of each sample, it is possible to determine the distance (Å) between the silica domains centers. The difference in the angle between each composition is minimal, always around the 6° corresponding to calculated distances between 14.0 and 14.7 Å. These values closely align with the 13 Å value previously calculated by Brus and Dybal for a similar hybrid system [73]. 

The textural properties of the materials were determined from N_2_ adsorption–desorption isotherms using the BET method (Figure 1d). The corresponding values of surface area, pore volume, and average diameter are detailed in Table 2. According to the IUPAC classification, the obtained isotherms for samples M0C0-I and M0C0-IV align with a type II isotherm, typically associated with nonporous or macroporous materials, exhibiting a hysteresis loop (type H3). As reported in the literature [74,75], this low-pressure hysteresis is attributed to the expansion of the non-rigid porous structure, resulting in a final adsorption curve value that does not correspond with the initial value. The isotherm for sample M0C0-III, on the other hand, corresponds to a type I isotherm, indicative of a material with a microporous structure. It is known from the literature that the acid/TEOS ratio influences both the crosslinking ratio, in the case of PDMS-TEOS hybrids, and the final porosity, as it influences the hydrolysis–condensation kinetics. A more acidic medium results in a more porous microstructure. The same applies to the water/TEOS ratio (*r*), with more porous microstructures being obtained when using a higher water/TEOS ratio up to 6. Above this value, the gelation kinetics slow down and a coarser microstructure is obtained [76,77].

### 3.2. Incorporation of RES

#### 3.2.1. Characterization of RES-Loaded Materials

**Figure 2:**
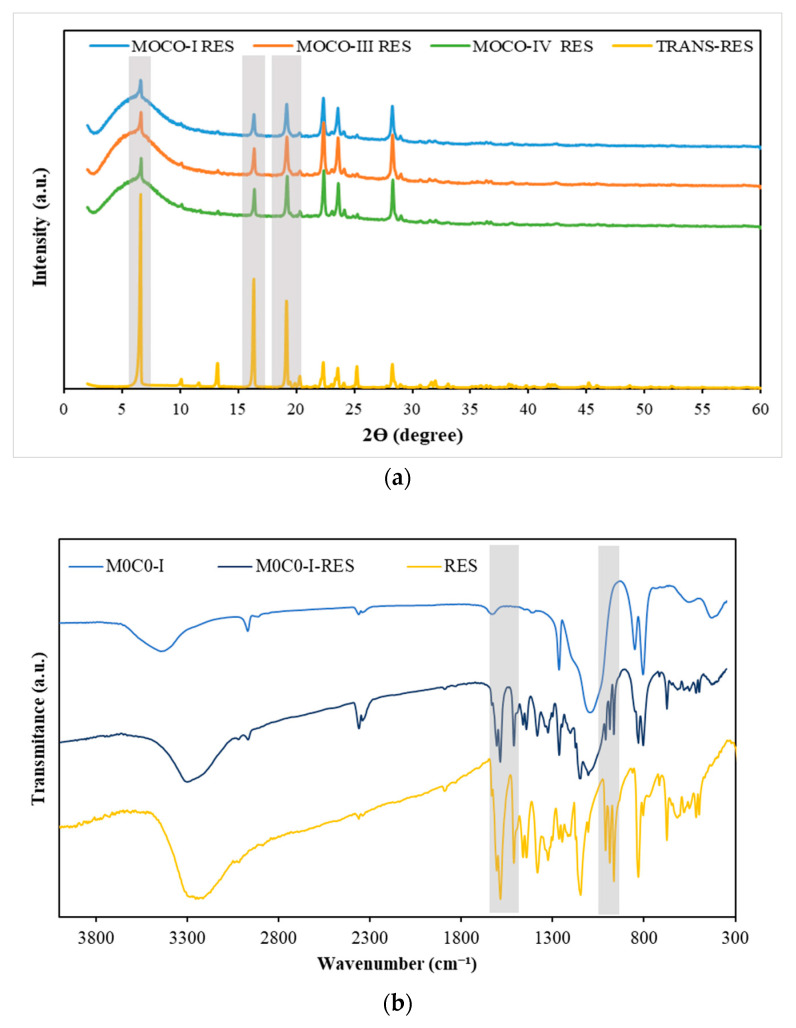
(**a**) XRD diffraction pattern of the PDMS-SiO2-RES samples and RES. (**b**) FTIR spectra in the interval of 300–4000 cm^−1^.

Loaded samples were further characterized by XRD and FTIR, aiming to understand whether RES was in the crystalline or amorphous state since this influences RES solubility and release. The powder XRD of free RES exhibited multiple sharp diffraction peaks between 10° and 30° due to the crystalline nature of the compound. The intensity of the peaks dropped in the diffractograms of the RES-loaded materials (Figure 2a), confirming that the loading of RES on the hybrids promoted the amorphization of the drug [57]. During the loading process by evaporation, RES molecules can be accommodated inside the pores or adsorbed at the surface of materials. The confinement of RES in pores may result in the amorphization of the drug, a phenomenon that is influenced by the pore size and surface chemistry of porous platforms materials [78,79]. Furthermore, some RES molecules may remain unbound, i.e., with no interaction with the material. Overall, the FTIR results (Figure 2b) align with those obtained from XRD analysis and further confirm the successful loading of RES into the materials. Major changes in the fingerprint region between 400 to 1700 cm^−1^ are clearly noticed when comparing the results of the loaded materials with the unloaded ones, marked by the appearance of several vibrational modes characteristic of the RES molecule. Notably, the band, centered at 1600 to 1400 cm^−1^, ascribed to the benzene skeleton vibrations and those between 989 and 964 cm^−1^ attributed to bending vibrations of C=C-H were observed [80,81]. On the other hand, the band associated with O-H stretching had shifted from 3430 to 3280 cm^−1^ in the loaded samples, indicating an interaction between the OH groups of the materials and RES. FTIR results were consistent across all the loaded materials.

**Figure 3:**
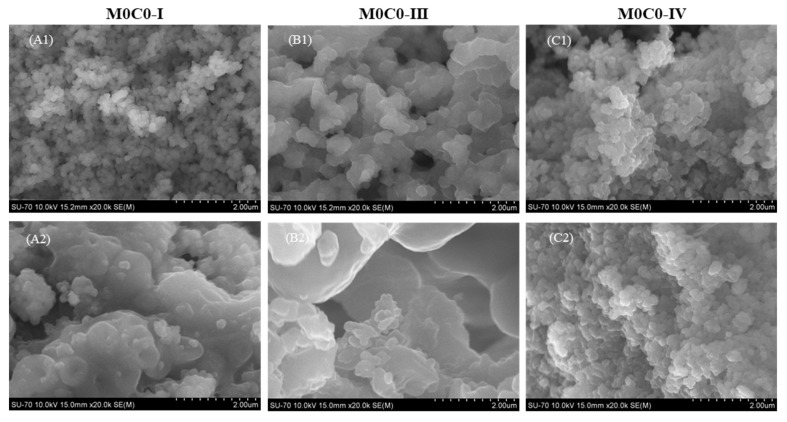
SEM micrographs of the unloaded and loaded samples: (**A1**) M0C0-I; (**A2**) M0C0-I-RES; (**B1**) M0C0-III; (**B2**) M0C0-III-RES; (**C1**) M0C0-IV; (**C2**) M0C0-IV-RES.

When compared with the powders before loading (Figure 3(A1,B1,C1)), microstructural changes were observed only in the M0C0-I-RES (Figure 3(A2)) and M0C0-III-RES (Figure 3(B2)) powders, despite the presence of resveratrol being noticeable in the structural analyses of all loaded powders. 

#### 3.2.2. Loading Capacity and Efficiency 

**Figure 4:**
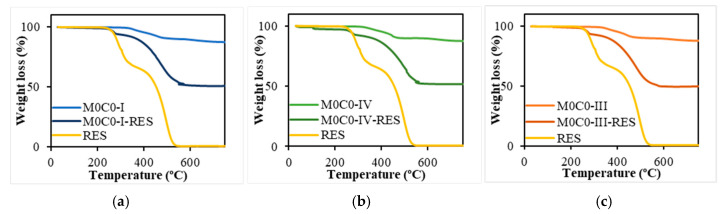
TGA analysis of unloaded and loaded PDMS-SiO_2_ samples and pure RES. (**a**) M0C0-I, M0C0-I-RES, and free RES; (**b**) M0C0-III, M0C0-III-RES, and free RES; (**c**) M0C0-IV, M0C0-IV-RES, and free RES.

TGA analysis was employed to determine the RES content in the loaded samples, i.e., the loading capacity (Figure 4). The unloaded materials presented a very small weight loss, with all samples losing approximately 12% of their initial weight around 750 °C, stabilizing for temperatures above 730 °C. This weight loss is attributed to the PDMS decomposition, more precisely to the formation of cyclic oligomers and their final decomposition [82]. In contrast, for RES-loaded materials, the weight loss was significantly higher, reaching around 51% at 700 °C, with curves stabilizing for temperatures above 600 °C. Regarding the RES thermogram, it is evident that weight loss occurs in two consecutive phases. The first, between 240 and 360 °C with a mass loss of 31.8%, and a second phase occurring approximately at 360–550 °C, with a weight loss of 66.6%, which is attributed to the oxidation of the carbonized material [83]. The curve stabilizes for temperatures above 550 °C, resulting in a total weight loss of 99.3%. The loading capacity was calculated by considering the weight loss observed for the loaded samples, excluding the weight loss contribution of the respective unloaded materials. The materials (200 mg) were loaded with an initial RES amount of 160 mg, so the theoretical loading capacity is 44.4%. The loading capacities and efficiencies are presented in Table 3. The theoretical and measured RES loading capacities were closely aligned, resulting in high loading efficiencies ranging from 75.9% to 85.4 %.

### 3.3. In Vitro Release Studies

**Figure 5:**
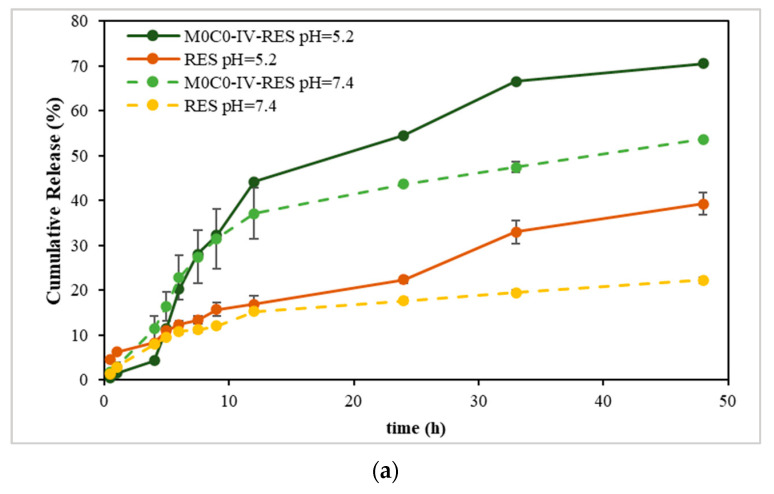

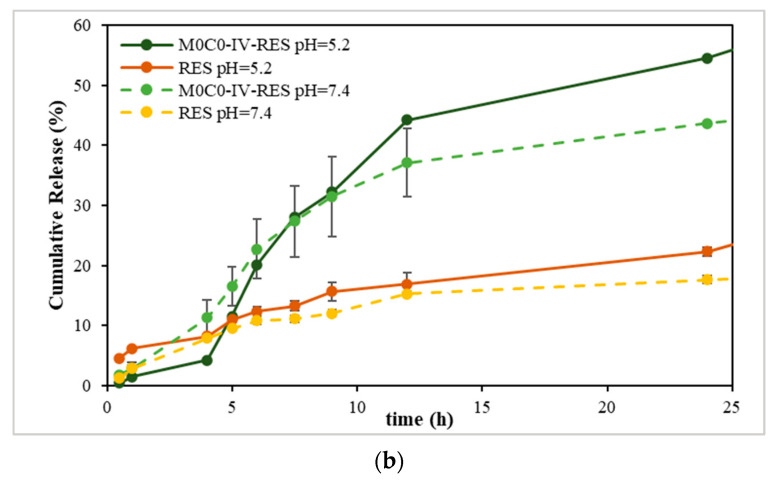
Cumulative release of RES from the non-encapsulated (free RES) and encapsulated sample (M0C0-IV-RES) at pH 5.2 and 7.4. (**a**) Full 48 h profile; (**b**) first 24 h profile.

The results obtained from the solubility assay (Figure S3; Supplementary Material), show that sample M0C0-IV-RES presented the highest concentration of dissolved RES, and considering this result, it was the sample chosen to proceed for the *in vitro* release studies. Figure 5 shows the cumulative release profiles of RES from the sample and free RES (non-encapsulated) at pH 5.2 and 7.4. Examining the release profiles, the cumulative release values at pH 7.4 after 48 h were 54% and 22% for sample M0C0-IV-RES and RES, respectively, increasing to 70% and 39%, respectively, at pH 5.2. Notably, RES release exhibited pH dependency, with a noticeably faster release observed at pH 5.2 compared to pH 7.4, evident for both M0C0-IV-RES and free RES. These findings align with prior studies on the stability and solubility of RES in various pH media, in which it was found that RES is more stable in acidic conditions, presenting higher values of solubility. Conversely, in alkaline conditions, RES tends to be more susceptible to degradation, resulting in lower solubility. This effect is likely due to concurrent degradation and dissolution mechanisms at higher pH values, leading to increased degraded products and the formation of insoluble complexes, thus decreasing RES solubility [58]. Additionally, it is worth noting that the loaded sample achieved higher cumulative release values over time at both pH values, compared to free RES, and demonstrated a gradual and sustained RES release without an initial burst. 

**Figure 6:**
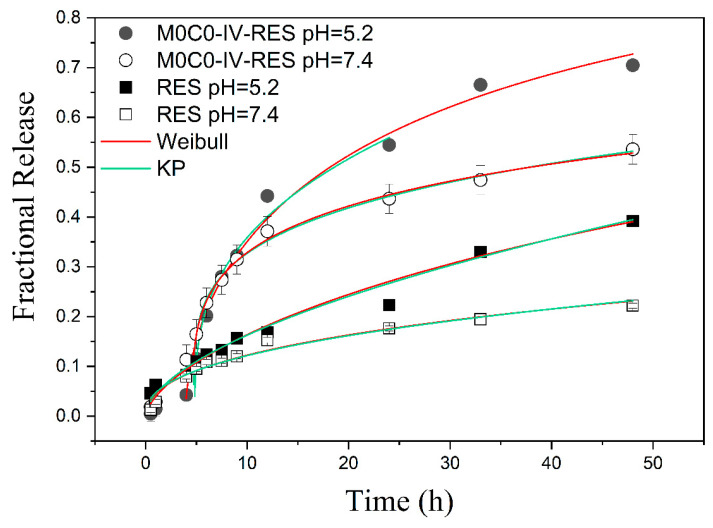
Fractional release of RES from the non-encapsulated (free RES) and encapsulated sample (M0C0-IV-RES) at pH 5.2 and 7.4, and fits using the Korsmeyer–Peppas (green line) and the Weibull (red line) models.

With regards to the kinetics and mechanisms of the release of RES, the NWF model demonstrated inadequate fit for all analyzed samples, implying non-Fickian diffusion. The Weibull model (described in Section 2.5.3) presents a better R^2^ fitting, nevertheless both Korsmeyer–Peppas and Weibull describe satisfactorily the experimental data points (Figure 6). For sample M0C0-IV-RES, the Korsmeyer–Peppas model revealed lag times (T*_i_*) of 4.8 h and 4.7 h at pH 5.2 and 7.4, respectively. The Weibull model yielded lag times of 3.9 h and 4.6 h for the same pH values. This indicates that the encapsulated RES release presents a delay of around 4.5 h until the onset of the dissolution process. In contrast, free RES exhibited no lag time in both pH conditions, indicating that this characteristic is intrinsic to the M0C0-IV-RES sample. This delay is likely attributed to the hydrophobicity of the sample, potentially hindering the release mechanisms, and resulting in a prolonged delay prior to the release of RES. From the Weibull model, the dissolution times, T*_d_*, were observed to be 27 h and 110 h for sample M0C0-IV-RES at pH 5.2 and 7.4, respectively, and 142 h and 868 h for free RES at the same pH values. These results further corroborate that the rate of RES dissolution is pH-dependent for both sample M0C0-IV-RES and RES alone and with the loaded material improving RES solubility and enabling a faster release rate. However, it is important to note that, as an empiric equation not deducted from kinetic fundamentals, the Weibull model does not provide specific insights into the mechanisms of RES release [63].

The values of *k*_KP_ obtained for sample M0C0-IV-RES were 0.20 and 0.22 h^−n^ at pH 5.2 and 7.4, respectively, and 0.043 and 0.047 h^−n^ for free RES at the same pH values. These values serve as indicators of the characteristics of the system, revealing changes when comparing free RES with our samples. Notably, there are no substantial changes in *k*_KP_ with variations in pH, suggesting that the system characteristics remain consistent for both M0C0-IV-RES and free RES. The values of *n* decrease with increasing pH for both M0C0-IV-RES and free RES, as illustrated in Table 4, meaning that the transport mechanism is pH-dependent [62,63].

### 3.4. Cell Viability in Osteosarcoma Cells

**Figure 7:**
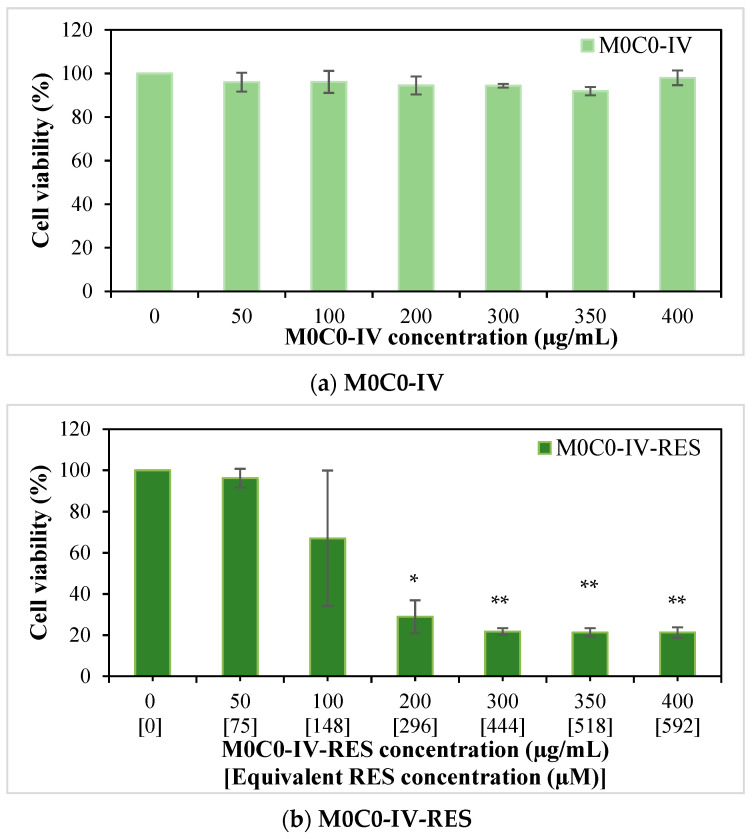

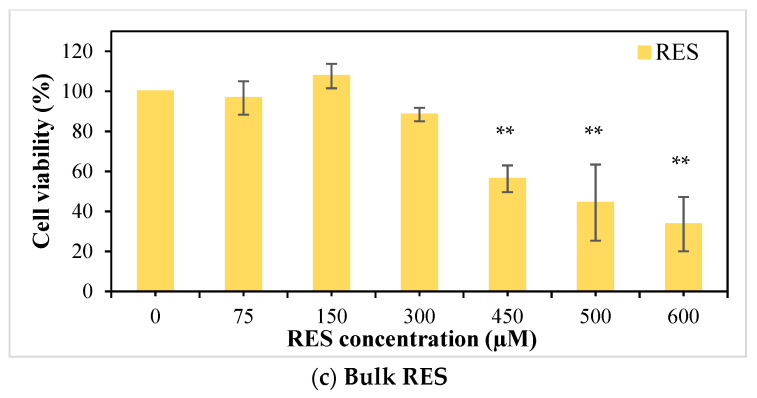
Effect of pristine M0C0-IV (0–400 µg/mL; (**a**)), RES-loaded M0C0-IV (0–400 µg/mL, (**b**)), and bulk RES (0–600 µM, (**c**)) on cell viability of MG-63 human osteosarcoma cells after 24 h of exposure. Results are expressed as mean ± SD, where * indicates statistically significant difference compared to the control with *p* < 0.05 and ** with *p* ≤ 0.001.

The cytotoxicity of RES-loaded hybrid (M0C-IV-RES), free RES, and pristine hybrid (M0C0-IV) on MG-63 osteosarcoma cells was evaluated using the MTT cell viability assay (Figure 7). The pristine hybrid did not affect cell viability, which was consistent with previous works regarding similar silica-based materials (Figure 7a) [84,85]. Bulk RES revealed a concentration-dependent cytotoxic effect (Figure 7c). For the highest concentration studied (600 µM), a percentage of cell viability of 33.6% was obtained. RES was pre-dissolved in DMSO; however, for cell treatment, this solution was diluted (1:100). The presence of DMSO did not affect cell viability (preliminary studies—data not shown). In sample M0C0-IV-RES, a gradual decrease in cell viability was noticeable with increasing concentration of the sample used (for concentrations > 50 µg mL^−1^), with a percentage of cell viability of 21.3% for the highest concentration of sample studied (400 µg mL^−1^), as seen in Figure 7b. However, when comparing these results with the ones from non-loaded RES, similar RES concentrations (in the loaded sample and bulk RES) gave rise to very different percentages of cell viability. In the case of the highest concentration of bulk RES studied (600 µM), a percentage of cell viability of 33.6% was obtained, while for a similar concentration of RES present in the loaded sample (592 µM), this value was 21.3%, and this trend is repeated for all studied RES concentrations higher than 75 µM. These results indicate that, for an equal time interval, there was a greater release of RES from the M0C0-IV-RES sample, consequently having a greater effect on cell viability when compared to RES alone, and further confirming the previous results obtained in the release studies.

## 4. Conclusions 

This work explored the PDMS-SiO_2_ system, combining its organic and inorganic components to create a material with adjustable properties. The polyphenol RES was effectively integrated into the hybrid materials, enhancing its solubility and release kinetics, particularly noticeable at pH 5.2. Mathematical analysis revealed the Weibull model as the best fit for describing RES release kinetics. The hybrid material exhibited an inherent initial time lag during release, unlike free RES. Cytotoxicity analysis on MG-63 cells showed no cytotoxicity for unloaded samples, while loaded samples exhibited decreased cell viability above 50 µg mL^−1^, with a notably higher effect compared to free RES. Overall, this study suggests significant potential for these materials in synthetic bone grafts and as drug delivery systems for RES.

## Figures and Tables

**Table 1:** Composition of the PDMS-SiO_2_ hybrid materials.

Sample	Composition (Molar Ratio/TEOS)			
	IPA	PDMS	H_2_O	HCl
M0C0-I	3.1	0.16	6.3	0.2
M0C0-II	3.1	0.16	6.3	0
M0C0-III	3.1	0.16	10.8	0.2
M0C0-IV	3.1	0.16	6.3	0.4

**Table 2:** Specific surface area values calculated from N_2_ adsorption isotherms for the PDMS-SiO_2_ samples.

	M0C0-I	M0C0-III	M0C0-IV
BET surface area (m^2^/g)	287.2	122.3	244.9
Pore volume (cm^3^/g)	0.990	0.121	0.890
Average pore diameter (nm)	168.9	3.47	99.82

**Table 3:** Values of loading capacity and efficiency for the RES PDMS-SiO_2_-loaded samples.

Sample	Loading Capacity (%)	Loading Efficiency (%)
M0C0-I-RES	36.4	82.0
M0C0-III-RES	37.9	85.4
M0C0-IV-RES	33.7	75.9

**Table 4:** Kinetics parameters and goodness of the fits of RES release from loaded materials and free RES. KP: Korsmeyer–Peppas.

Model		M0C0-IV-RES		RES	
		pH 5.2	pH 7.4	pH 5.2	pH 7.4
**KP**	***k*_KP_** (h^−n^)	0.20 ± 0.02	0.22 ± 0.04	0.044 ± 0.003	0.047 ± 0.005
	***T_i_***	4.8 ± 0.2	4.7 ± 0.4	0	0
	***n***	0.34 ± 0.05	0.23 ± 0.05	0.57 ± 0.02	0.41 ± 0.03
	**R^2^**	0.9668	0.8908	0.9896	0.9625
	**χ^2^**	0.0010	0.0022	0.0001	0.0002
**Weibull**	**α** (h^β^)	6.3 ± 0.6	4.1 ± 0.9	25 ± 2	21 ± 2
	**β**	0.56 ± 0.03	0.30 ± 0.07	0.65 ± 0.01	0.45 ± 0.03
	**T*_i_*** (h)	3.93 ± 0.09	4.6 ± 0.6	0	0
	**T*_d_*** (h)	27 ± 1	110 ± 9	142 ± 30	868 ± 74
	**R^2^**	0.9919	0.9533	0.9782	0.9670
	**χ^2^**	0.0007	0.0018	0.0003	0.0002

## Data Availability

All data reported in this paper are contained within the manuscript and Supplementary Materials.

## Supplementary Materials

The following supporting information can be downloaded at https://www.mdpi.com/article/10.3390/polym16070879/s1, Figure S1: Photographs of the obtained materials after being dried and before being grounded to powder; Figure S2: Flowchart of the synthesis procedure and post synthesis treatments; Figure S3: Solubility profiles of RES and RES-loaded materials at pH 7.4 after 24 h.
