# Supplementary material for: Resveratrol-Loaded Polydimethylsiloxane–Silica Hybrid Materials: Synthesis, Characterization, and Antitumoral Activity
Source: Polymers (Basel). 2024 Mar 22;16(7):879. doi: 10.3390/polym16070879 (PMC11013690; doi:10.3390/polym16070879)
Supplement: Supplementary file 1 [file polymers-16-00879-s001.zip › polymers-2884449-supplementary.pdf]

Resveratrol-Loaded Polydimethylsiloxane–Silica Hybrid Materials: Synthesis, Characterization, and Antitumoral Activity

Sofia Viegas [1], Diogo Marinheiro [1], Verónica Bastos [2], Ana L. Daniel-da-Silva [1],
Ricardo Vieira [1], Helena Oliveira [2], José Carlos Almeida [3,*] and Bárbara J. M. L. Ferreira [1,*]

[1] Department of Chemistry, CICECO-Aveiro Institute of Materials, University of Aveiro, 3810-193 Aveiro, Portugal

[2] Department of Biology, CESAM-Centre for Environmental and Marine Studies, University of Aveiro, 3810-193 Aveiro, Portugal

[3] Department of Materials and Ceramic Engineering, CICECO-Aveiro Institute of Materials, University of Aveiro, 3810-193 Aveiro, Portugal

* Corresponding author e-mail address: jcalmeida@ua.pt; barbaraferreira@ua.pt

Supplementary material

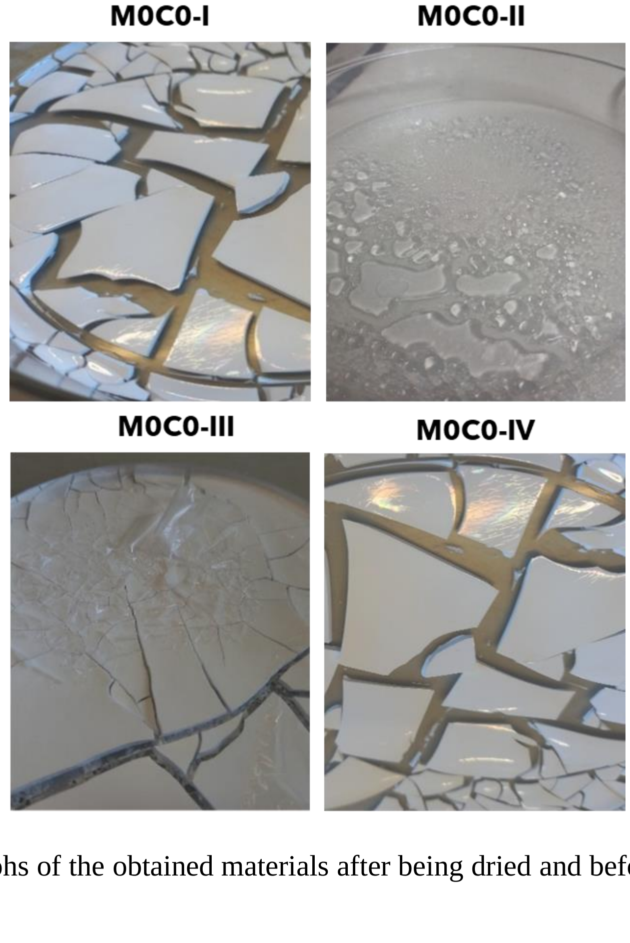

Figure S1: Photographs of the obtained materials after being dried and before being grounded to powder.

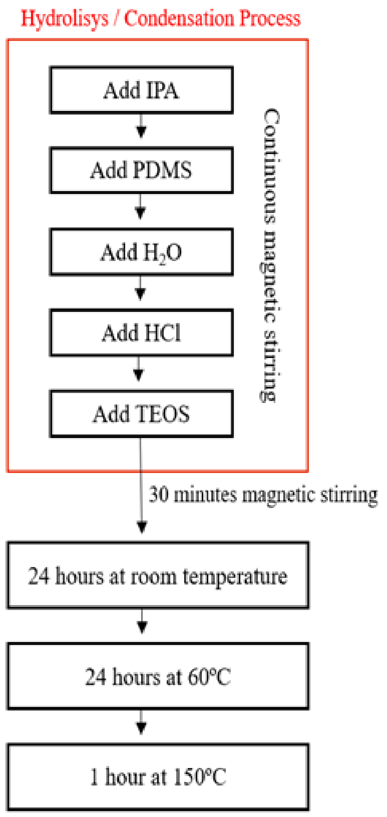

Figure S2: Flowchart of the synthesis procedure and post synthesis treatments.

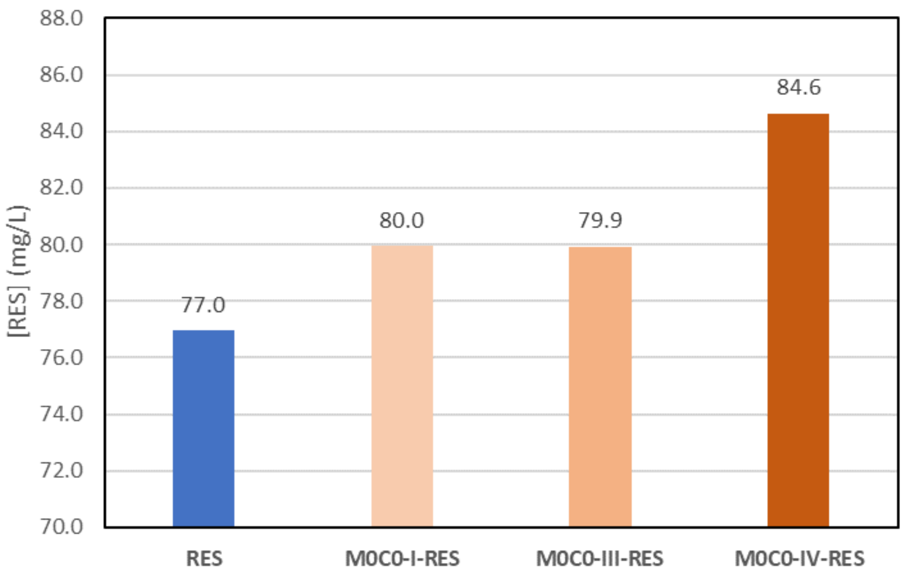

Figure S3: Solubility profiles of RES and RES loaded materials at pH 7.4 after 24 hours.
